# Brain Reorganization in Patients with Brachial Plexus Injury: A Longitudinal Functional MRI Study

**DOI:** 10.1100/2012/501751

**Published:** 2012-05-01

**Authors:** Takeharu Yoshikawa, Naoto Hayashi, Yasuhito Tajiri, Yoshirou Satake, Kuni Ohtomo

**Affiliations:** ^1^Department of Computational Diagnostic Radiology and Preventive Medicine, The University of Tokyo Hospital, 7-3-1 Hongo, Bunkyo, Tokyo 113-8655, Japan; ^2^Department of Orthopedic Surgery, Tokyo Metropolitan Hiroo Hospital, 2-34-10 Ebisu, Shibuya, Tokyo 150-0013, Japan; ^3^Department of Radiology, The University of Tokyo Hospital, 7-3-1 Hongo, Bunkyo, Tokyo 113-8655, Japan

## Abstract

The aim of this study is to assess plastic changes of the sensorimotor cortex (SMC) in patients with traumatic brachial plexus injury (BPI) using functional magnetic resonance imaging (fMRI). Twenty patients with traumatic BPI underwent fMRI using blood oxygen level-dependent technique with echo-planar imaging before the operation. Sixteen patients underwent their second fMRI at approximately one year after injury. The subjects performed two tasks: a flexion-extension task of the affected elbow and a task of the unaffected elbow. After activation, maps were generated, the number of significantly activated voxels in SMC contralateral to the elbow movement in the affected elbow task study (*N*_af_) and that in the unaffected task study (*N*_unaf_) were counted. An asymmetry index (AI) was calculated, where AI = (*N*_af_ − *N*_unaf_)/(*N*_af_ + *N*_unaf_). Ten healthy volunteers were also included in this fMRI study. The AI of the first fMRI of the patients with BPI was significantly lower than that of the healthy subjects (*P* = 0.035). The AI of the second fMRI significantly decreased compared with that of the first fMRI (*P* = 0.045). Brain reorganization associates with peripheral nervous changes after BPI and after operation for functional reconstruction.

## 1. Introduction

 Brachial plexus injury (BPI) is caused by accidental traction of the upper limb, mainly in a motorcycle accident, resulting in complete or partial motor paralysis [1]. The treatment method for brachial plexus injury varies, depending on the degree of pathological condition and the location of the damaged site [1–4]. If elbow flexion is impaired due to preganglionic damage, intercostal nerve transfer may be performed to restore motor function [5–7]. Favorable treatment outcome is obtained in 82% of such cases when the patient is under 40 years of age, and the intercostal nerve transfer procedure is done within 6 months of the brachial plexus injury [8].

 Intercostal nerves basically regulate muscles related to respiration and posture control. After operation of intercostal nerve transfer, the role of the transferred nerve can gradually be transformed into the new pathway for regulation of elbow flexion. This occurs as a result of the central nerve system's adaptation to the alteration of the peripheral nerve connection. There have been only a few papers regarding associated changes in the brain, namely, brain reorganization, to cope with such alteration in patients who have undergone treatment for brachial plexus injury [9–16]. Moreover, these prior studies evaluated the condition only at a certain point in time or involved a small number of patients; there have been no reports on serial changes of the brain in a longitudinal followup.

 Functional magnetic resonance imaging (fMRI) using blood oxygen level-dependent effect, because of its excellent spatial resolution [17, 18], has been clinically applied to evaluate brain recovery, including the observation of a specific site of the brain during the process of motor function recovery after cerebral infarction. Researchers have reported on fMRI comparative findings on stroke patients and normal controls [19–23], and on sequential changes over a certain period [24–26]. However, follow-up studies have usually been done within a 6-month period, since recovery from cerebral infarction has generally been considered to occur within 6 months after treatment [27]. And these prior studies have tended to be limited to mild or recovering cases [28]. In other studies on the central nervous system, fMRI has been used to evaluate plasticity of the brain in patients with multiple sclerosis [29], cerebral autosomal dominant arteriopathy with subcortical infarcts and leukoencephalopathy [30], and arteriovenous malformation [31], although these cases did not include long-term evaluations.

 Studies using fMRI have also included conditions other than cerebral disorders, such as brachial plexus injury [14–16], patients undergoing amputation [32–34], neuropathy [35], and spinal cord injury [36], all covering limited period of time. Malessy et al. reported that fMRI showed no brain activation with a mental motor task of the paralyzed arm prior to treatment [16], suggesting limited brain function, that is, an impaired neural input/output pathway due to peripheral nerve injury. They also reported that cerebral activation equal to that of the contralateral unaffected hemisphere was seen regulating the motor function of the arm at 45 months or later after treatment [16], reflecting changes in the brain after recovery of neural input/output activity. However, they did not show data of sequential changes in fMRI of one patient or on comparative findings before and after treatment.

 The fact that restriction and recovery of neural input/output activity occurs over several months in patients with brachial plexus injury suggests a multistage brain reorganization process, corresponding to the changes in neural input/output activity. It is presumed that the degree of activation related to the motor function of patient's limbs decreases until the recovery of neural input/output activity, and increases later, although no data have been reported that prove this assumption. Another confusing factor is the alteration of volitional muscle control nerves before and after intercostal nerve transfer. To clarify the complex long-term brain reorganization process, sequential follow-up study on the same patient is essential.

The purpose of our study was to observe serial fMRI findings of brain reorganization in patients with paralysis due to brachial plexus injury, with a special emphasis on the changes in motor cortex activation contralateral to the elbow movement of the affected elbow, and on the reactivation after treatment over a long-term functional recovery period.

## 2. Materials and Methods

### 2.1. Subjects

 Our subjects were 20 patients with brachial plexus injuries who had been hospitalized in the Orthopaedic Department of our institution for further examination and treatment. The diagnosis of brachial plexus paralysis, the site of brachial plexus injury, and the extent of paralysis and pathological condition were determined by certificated orthopaedic surgeons, based on consultation, electrophysiologic examination, imaging examination, and intraoperative findings. The patients included 19 males and one female ranging from 18–41 years of age with an average age of 25.1 ± 6.8 years (mean ± SD). All were right-handed individuals. Among them, 14 had a right-side injury, and six had a left-side injury. Nine experienced complete paralysis, while the remaining 11 presented with partial paralysis. Upon admission into our hospital, 16 patients could not volitionally flex their elbow, while four could. Manual muscle test (MMT) of the biceps brachii muscle of the affected side was zero or 1 in 17 of the patients and 2 or higher in the other three patients. One patient was able to flex his elbow even though the muscle force was zero, because the brachioradial muscle force registered a score of 4. Neither injury nor other disease was found in the brains of the subject patients.

 All patients underwent surgery for functional reconstruction; 15 patients underwent nerve transfer, and five patients underwent nerve grafting. The time from the injury to the surgery ranged from 1 to 5 months (mean 2.7 ± 1.2 months). In patients who undergoing the surgical exploration and the functional reconstruction on different days, the day for the functional reconstruction was defined as the day of surgery. The clinical features of the 20 patients are summarized in Table 1. After discharge from our hospital, patients were monitored through the outpatient clinic. Nine patients were followed up for 2 or more years. According to the MMT, the score of the muscle force of the biceps brachii muscle of one patient was 5 (before operation) and that of seven patients improved from zero or 1 to 2 or higher on the last physical examinations. One patient did not show good recovery.

 All of the 20 patients underwent their first fMRI examination preoperatively during hospitalization at zero to 5 months after injury (average 2.0 ± 1.3 months). Follow-up fMRI examinations were scheduled in most cases at approximately one year, two years, and three years after injury. Sixteen patients underwent their second fMRI examination at 9 to 14 months after injury (average 12.4 ± 1.5 months), which was 7 to 12 months after operation (average 9.1 ± 1.3 months). Nine patients underwent their third fMRI examination at 20 to 24 months after injury (average 22.3 ± 1.5 months), which was 17 to 20 months after operation (18.6 ± 1.4 months). Four patients underwent their fourth fMRI examination at 33 to 35 months after injury (average 34.0 ± 0.8 months), which was 31 to 32 months after operation (31.3 ± 0.5 months).

 As a healthy control group, 10 volunteers underwent fMRI under the same conditions. They included 9 males and one female ranging from age 28–42 years of age, with an average of 35.1 ± 5.2 years (mean ± SD). All were right-handed individuals.

All subjects, including the BPI patients group and the healthy control group, were fully informed about the experiment procedures and gave a written form of consent. Approval from the Ethics Committee at our institution was obtained for this study. 

### 2.2. Motor Task and Paradigm

The motor task for fMRI was repetitive flexion/extension of the right and left elbows. When patients were unable to execute this movement volitionally, they were encouraged virtually rather than literally to perform the task with the intension to move as much as possible. Patients with partial paralysis who had limitation of the flexion angle or muscle force were also required to attempt movement in the same manner. The patients and the control group were asked to move their healthy arm to the greatest extent within an unobstructed range in the MRI gantry on the scanning table. The tasks were performed after a detailed explanation was given to the individual before entering the MRI examination room, and subjects practiced on the scanning table immediately before the initiation of the MRI examination to make sure that they understood the task. During the examination, the motor task and presence or absence of associated movements and/or a mirror movement were visually monitored by the examiner. When the actual motor task was considered unsatisfactory, reexamination was conducted. After the MRI examination, patients were asked to reconfirm that they carried out the motor task as much as possible.

The fMRI paradigm was a box-car type block design. The movement task was presented in 30-second movement blocks alternating with 30-second rest periods for a total of four paired blocks. The subjects were instructed to “Move (ON)” and “Stop (OFF)” the arm movement at the onset of the respective time periods by the examiner using an intercommunication device.

### 2.3. fMRI Imaging Technique

Images were obtained using a 1.5T MR unit (Signa Horizon LX 1.5T ver 8.25/8.3/9/11.0, GE Medical Systems, Milwaukee, WI) with a standard quadrature head coil. The head of subjects was fixed on the scanning table using a pad to minimize shift due to body motion. Patients were asked not to make any body motions other than those they were instructed to do.

Axial images of the whole brain were obtained with gradient-echo echo-planar sequence, which is sensitive to the blood oxygen level-dependent contrast. The fMRI imaging parameters were set at repetition time 3000 msec, echo time 50 msec, flip angle of 90 degrees, slice thickness of 7 mm, inter-slice gap of 1 mm, field of view of 24 × 24 cm^2^, with a 64 × 64 imaging matrix, a total of 18 slices, number of excitations of 1, and a band width at 62.5 kHz.

Although we initially set up the scanning time for one series as 4 minutes and 30 seconds, images scanned in the first 30 seconds were excluded due to unstable magnetic uniformity immediately after initiation of scanning, and only the other 80 images obtained in the last 4 minutes were analyzed in our study.

After completing the fMRI, three-dimensional T1-weighted images of the whole brain were obtained at fast SPGR (fast spoiled gradient recalled acquisition in steady state) sequence. The imaging parameters of T1-weighted image were set at repetition time 200 msec/echo time 1.9 msec, flip angle of 20 degrees, slice thickness of 1.4 mm, field of view of 24 × 24 cm^2^, with a 256 × 256 × 128 imaging matrix, number of excitations of 2, and a band width of 15.63 kHz. The total scanning time was 9 minutes 50 seconds.

### 2.4. Statistical Parametric Mapping (SPM) Analysis

Statistical Parametric Mapping (SPM) (Welcome Department of Imaging NeuroscienceInstitute of Neurology, University College London, London, UK) is a freely available software to the neuroimaging community and includes brain functional images [37, 38]. We used SPM99 which is operated with MEDx 3.3 on a workstation (Sun Ultra 10, Sun Microsystems, Santa Clara, CA) and SPM99 which is operated by MALTAB 6.5 on Windows PC.

 All acquired images of fMRI were transferred to the Sun Ultra 10 workstation and were transformed into “Analyze file format” (Biomedical Imaging Resource, Mayo Clinic, Rochester, MN). Following this, as spatial preprocessing by SPM, realignment [39], spatial normalization, and smoothing using a Gaussian filter of a 6 mm full-width at half-maximum were done [40].

Changes of MR signals were evaluated by defining a design matrix that corresponded with the scanning parameters for image acquisition. The presence of a significantly activated voxel was defined when it exceeded the significance level of *P* < 0.05 with multiple comparison correction, and maximum intensity projection images were processed to reflect the activated region. Anatomical location of the activated voxels was defined using enhanced SPM software, “MNI Space Utility” (Positron Emission Tomography Lab of the Institute of the Human Brain, St. Petersburg, Russia, URL: http://ihb.spb.ru/~pet_lab/MSU/MSUMain.html), and the number of activated voxels in the sensorimotor cortex (SMC) of bilateral hemispheres was counted.

 In order to evaluate activation of the SMC contralateral to the affected side relative to that of the other side, an asymmetry index (AI) was calculated. The AI was defined based on the following formula, supposing activation of the SMC contralateral to the unaffected side is almost the same between BPI patients and healthy subjects during a motor task of the unaffected elbow [12]:

(1) $$\text{AI}=\frac{N_{\text{af}}-N_{\text{unaf}}}{N_{\text{af}}+N_{\text{unaf}}},$$

where *N*_af_ is the number of activated voxels in the SMC contralateral to the affected side during the motor task of the affected side, and *N*_unaf_ is the number of activated voxels in the SMC contralateral to the unaffected side during the motor task of the unaffected side.

On the other hand, the AI of healthy subjects was calculated by the following formula:

(2) $$\text{AI}=\frac{N_{\text{right}}-N_{\text{left}}}{N_{\text{right}}+N_{\text{left}}},$$

where *N*_right_ is the number of activated voxels in the SMC of the left side during the motor task of the right side, and *N*_left_ is the number of activated voxels in the SMC of the right side during the motor task of the left side.

 In order to clarify the difference between the preoperative BPI patients with disturbance of elbow flexion/extension and healthy subjects, the AI at the patient's first fMRI and the AI of healthy subjects were compared. To eliminate any possibility of confusion over distinction between right and left-side differences, we compared 11 patients with right-side injury only with 10 healthy subjects. The AIs of the two groups were compared with Student's *t*-test.

 To evaluate changes occurring in BPI patients from before their operation to 9 months after operation, the AI's of the first and the second fMRI were compared in the 13 patients with disturbance of elbow flexion/extension at admission. The AIs of the two groups were compared with paired Student's *t*-test.

Analysis based on a fixed-effect model was conducted on 7 of 8 patients who had a disturbance of elbow flexion/extension at admission and underwent a third fMRI. One patient showing no signs of recovery was excluded from this analysis. Results of the motor task of the affected side for the first, second, and third fMRIs were analyzed.

We also conducted a fixed-effect model analysis on 3 of 4 patients who had disturbance of elbow flexion/extension at admission and who underwent a fourth fMRI, again excluding one patient showing no recovery. Data of the results of the motor task of the affected side for the first, second, third, and fourth fMRI were analyzed. Using the MNI Space Utility of the SPM software, the number of activated voxels was counted in the SMC of both hemispheres. For reference, the same analysis method was applied to evaluate the motor task of the unaffected side.

## 3. Results

We studied brain reorganization in BPI patients sequentially using fMRI. In BPI patients with disturbance in flexion/extension motion of the elbow, activation in the SMC contralateral to the affected side decreased at approximately 3 months after injury. And it was even more minimized at one year after injury (approximately 9 months after surgery). Eventually, in accordance with the recovery of flexion/extension ability of the elbow, activation of the SMC contralateral to the affected side is considered to recover.

All 20 patients underwent their first fMRI examination successfully during hospitalization, and 16 patients underwent a second fMRI examination, 9 patients a third fMRI examination, and 4 patients a fourth fMRI examination at ambulatory. There were no problems in the achievement of the task and image acquisition for all subjects, although some of them showed slight associated movement of the shoulder, wrist, hand, or leg (5 of 20 patients in the first fMRI examination, 5 of 16 in the second fMRI, and 2 of 9 in the third fMRI). No mirror movement was observed. fMRI was performed on 10 healthy volunteers as scheduled without any problem.

**Figure 1:**
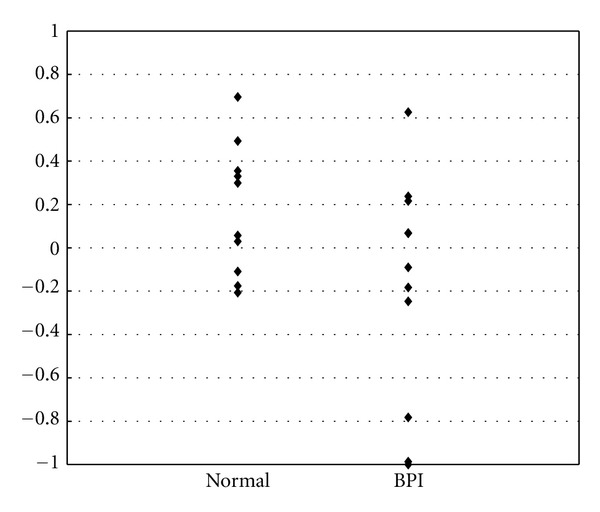
AI (asymmetry index) at the first fMRI of patients with right side BPI (brachial plexus injury) as compared with normal subjects.

Figure 1 demonstrates the AI at the first fMRI of patients with right-side BPI and impaired flexion/extension movement at admission as compared with healthy subjects. The AI of the patients was significantly lower than that of the healthy subjects (*P* = 0.035).

**Figure 2:**
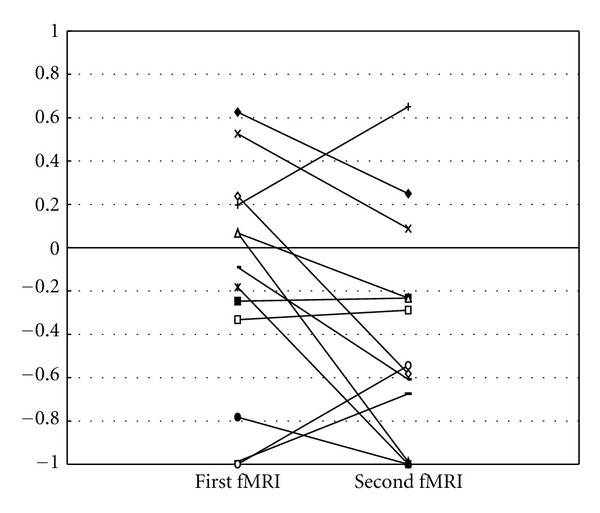
A comparison of AI between the first fMRI and the second fMRI of patients with BPI.

Figure 2 shows a comparison of AI between the first fMRI and the second fMRI of patients with BPI and impaired flexion/extension movement at admission. The second AI of these patients significantly decreased compared with the first AI (*P* = 0.045).

**Figure 3:**
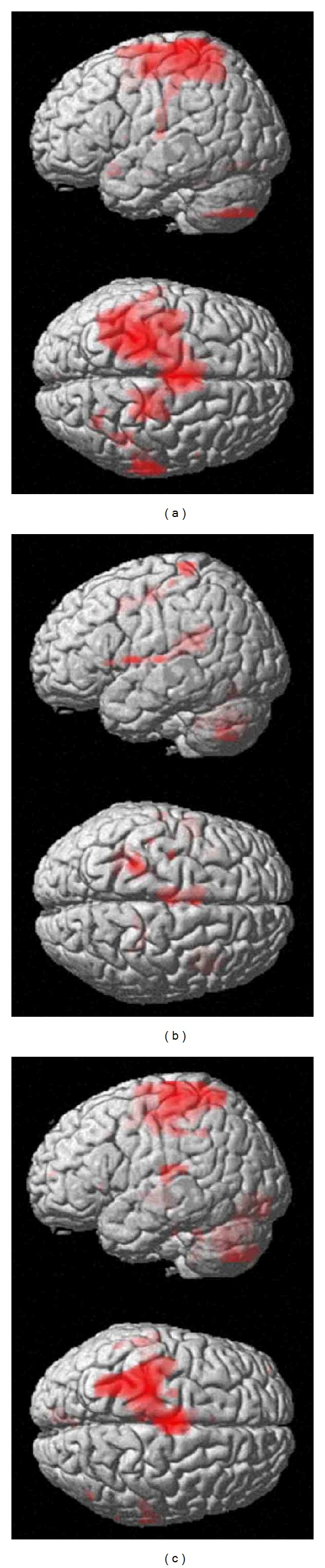
A rendering of analysis results using the fixed-effect model of seven BPI patients during the motor task of the affected elbow. (a) the first fMRI (before operation), (b) the second fMRI (approximately 1 year after operation), and (c) the third fMRI (approximately 2 years after operation).

Figure 3 demonstrates a rendering of analysis results using the fixed-effect model of seven patients during the motor task of the affected side in serial fMRI examinations. Activation of the contralateral SMC decreased at the second examination, but increased at the third examination.

**Figure 4:**
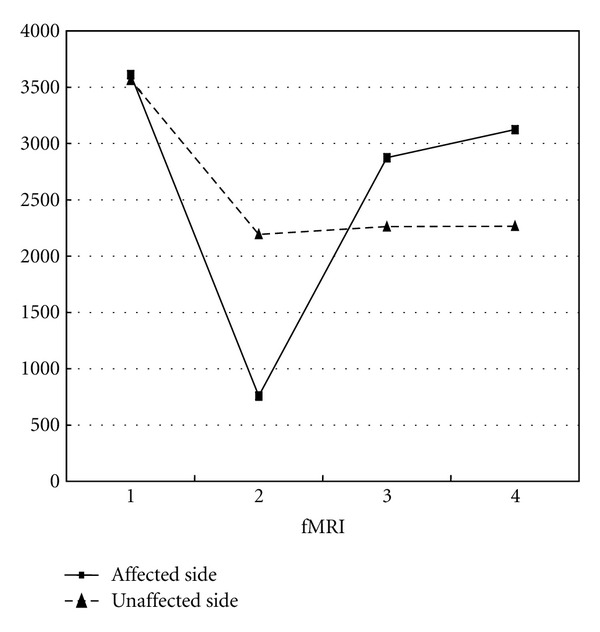
A transition of the number of activated voxels in the contralateral SMC of three patients with good recovery.

Figure 4 shows a transition of the number of activated voxels in the contralateral SMC of three patients who had good recovery, fMRI with motor task from the first to the fourth examination of the affected side and unaffected side. The activation of the contralateral SMC decreased at the second fMRI examination but tended to recover at the third and fourth fMRI examinations.

**Figure 5:**
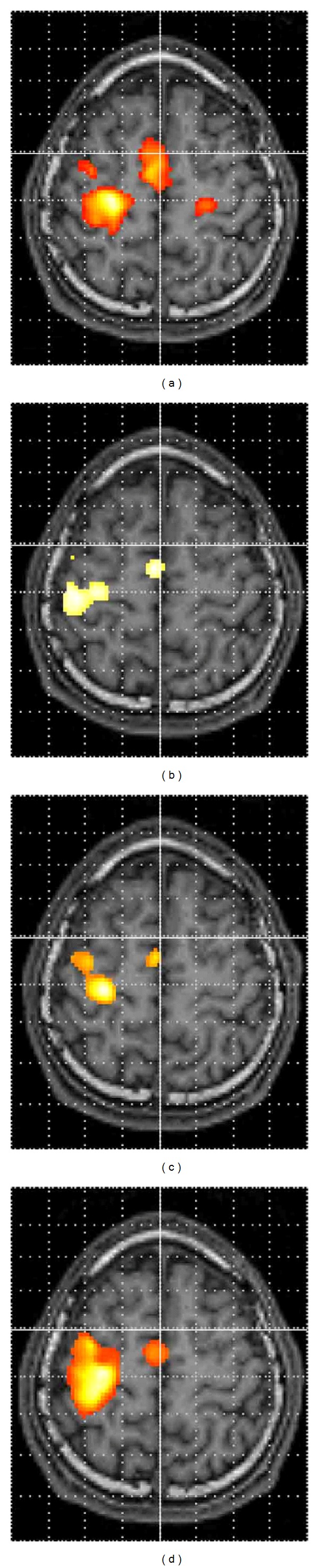
Serial fMRI results of a 18-year-old male right BPI patient with good recovery. (a) The first fMRI (before operation), (b) the second fMRI (approximately 1 year after operation), (c) the third fMRI (approximately 2 years after operation), and (d) the fourth fMRI (approximately 3 years after operation). The patients' right is on the observers' right.

Figure 5 shows serial fMRI results during the motor task of the affected side of a right BPI patient. The activation of the contralateral SMC decreased at the second fMRI examination but tended to recover at the third and fourth fMRI examinations. His biceps brachii muscle force improved to 3 on MMT 3 years after operation.

**Figure 6:**
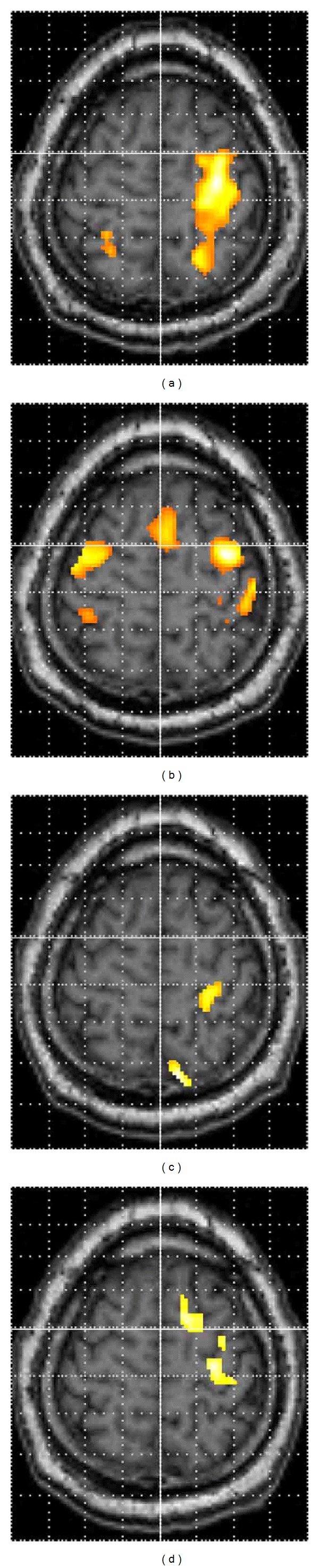
Serial fMRI results of a 34-year-old male with left BPI during the motor task of the affected elbow. (a) The first fMRI (before operation), (b) the second fMRI (approximately 1 year after operation), (c) the third fMRI (approximately 2 years after operation), and (d) the fourth fMRI (approximately 3 years after operation). The patients' right is on the observers' right.

Figure 6 shows serial fMRI results during the motor task of the affected side of a left BPI patient. The activation of the contralateral SMC continued at a low level through the fourth examination. His elbow movement had no favorable recovery.

## 4. Discussion

The comparison between the AI at the first fMRI of patients with impaired flexion/extension movement and that of healthy subjects showed that the preoperative patients had less brain activation in the SMC contralateral to the affected side, compared with the SMC contralateral to the unaffected side. Supposing the activation of the SMC contralateral to the unaffected side should be almost equal between the BPI patients and healthy subjects [12], activation of the SMC contralateral to the unaffected side of preoperative patients is considered less than that of the healthy subjects.

Our results concurred with those of previous studies. The pathological condition of patients undergoing amputation is similar to BPI patients, insofar as the fact of the connection between the central nerve system and peripheral nerves is being impaired. The fMRI of an imaginary motor task of a phantom limb of an amputee patient showed activation in the SMC contralateral to the amputated side, symmetrical to that of the unaffected side, and distribution of activation was less in the SMC contralateral to the amputated side than in the unaffected side [32, 34]. These results agreed with those of our study findings. Considering the fact that SMC of healthy subjects shows activation with such imaginary motor task, even without any physical movement, but the activation is less than that with actual motor task [41], the result must be reasonable. Malessy et al. reported that the fMRI of two BPI patients, prior to undergoing surgery for functional reconstruction, showed no activation in connection with imaginary motor task of the paralyzed elbow [16]. It agrees with our study in terms of the reduced activation; however, considering the fact the activation occurs with imaginary motor task in amputation patients or healthy subjects, it is contradictory that there two patients showed no activation. That may be because they happened to have studied on patients with weak activation, or there was a difference in imaging data acquisition or data processing between their study and ours.

 There is a report regarding patients who received amputation of upper limb from early on had more brain activation during anteflexion movement of the stump than that of the unaffected limb, presumably because disinhibition occurred in the cortex [33]. This was logically consistent when there was a contraction of cortical region corresponding to the movement of amputated upper limb, an enlargement of other cortical region connected to the movement of the area adjacent to the amputated site occurred. Similarly, research of patients with paraplegia due to spinal cord injury showed an enlarged cortical region for finger motion control [36]. Whereas, Reddy et al. reported fMRI of neuropathy patients had enlarged activation in the motor cortex contralateral to the side of motor task [35]; however, probably there was a difference in pathological condition compared to our study because their patients could achieve the assigned motor task.

In order to avoid including influence of dominant arm on activation of right and left hemispheres, we selected right side injury of right-handed subjects only in the comparison of AI of the first fMRI examination and that of healthy subjects. Further investigation with more number of patients including those with left-side injury would be necessary.

 We calculated the AI from the number of activated voxels in the motor cortex contralateral to the affected side during the motor task of the affected side, and those in the unaffected side during the motor task of the unaffected side. Therefore, we could reduce dispersion between the subjects and could evaluate relative activation changes. Whereas, in studies of patients with cerebral infarction, a “laterality index” is sometimes used. The laterality index is applied in the evaluation of patients with cerebral infarction, calculating the ratio between the number of activated voxels in the SMC contralateral to the motor task side and those ipsilateral to the motor task side [19, 21, 23, 24, 26, 29, 30]. The laterality index is used when comparing differences of involvement of the contralateral hemisphere and ipsilateral hemisphere on the same motor task. In this study, we did not use the laterality index, but we used AI.

Comparisons of the patients' AI of the first and the second examinations indicated that activation of the SMC contralateral to the affected side decreased one year after injury (second examination) relative to that of three months after injury (first examination). The cause for this decrease is believed to be related to the following mechanism. When there are no neural input/output signals, activation of the SMC related to the contralateral elbow flexion movement (affected side) will not increase, but may decrease. At the second examination (one year after injury), the route connecting the volitional elbow flexion and the biceps brachii muscle has not yet been established, and leaving it difficult for the patient to contract the biceps brachii muscle without voluntary respiration. Therefore, regardless of the patient's efforts to flex the elbow, a greater level of activation than at the first examination remained impossible in the second examination. The natural recovery of reduced muscle force is sometimes observed within three months after injury, but not after that. These facts support our findings that SMC activation increased within 3 months of injury, owing to hyperexcitability, elimination of disinhibition, or compensatory movements to assist functional recovery, but decreased after 3 months.

 The analysis based on the fixed-effect model during the motor task of the affected side from the first to the third fMRI examinations showed that the activation of the contralateral SMC decreased at the second fMRI examination, but tended to recover by the third fMRI examination. Also, transition of the number of activated voxels in the contralateral SMC during the motor task of the affected side (from the first to the fourth fMRI examination) in patients who had recovery showed a reduced number of activated voxels at the second examination and a subsequent increase at the third to fourth examination. Malessy et al. performed fMRI on recovering patients from 45 to 103 months after BPI surgery [16]. They reported that during elbow flexion task, activation in the primary motor cortex contralateral to the affected side did not differ significantly from that contralateral to the unaffected side. It is known that muscle force recovery continues for several years after suture of the peripheral nerves. Therefore, we predict that our patients will show continuing recovery of muscle force and activation in the SMC to a point close to normal level.

 One of our patients showed unfavorable recovery progress as of 31 months after surgery. The transition of fMRI of this patient indicated activation of the SMC contralateral to the affected side before the operation; however, the activation continued at a low level through the fourth examination. While the key indicator-site of nonrecovery has yet to be pinpointed (either the muscle, peripheral nerve, spinal cord, or brain), it is considered that the activation of the SMC would not improve but rather be minimized when the muscle force is not recovered. Also it may be possible to consider that the role of adjacent region would be expanded, such that in patients undergoing amputation of the upper limb, the role of specific motor cortex corresponding to the movement of the stump was enlarged [33].

 The motor task of our fMRI examination was a flexion/extension movement of the elbow. When the motor function of the upper limb is impaired due to BPI, the prior function to reconstruct is elbow flexion [1, 7]. Previous studies of fMRI on BPI patients also applied elbow flexion as a motor task [14, 16].

 Homology of the task is important in fMRI study on motor function. In our study, when motor function of patients was impaired, they were asked to have the intention to flex their elbow as strongly as possible. It is known that an enlarged activated area in the brain is generally observed in accordance with more greatly evoked muscle strength in fMRI during motor task. On the other hand, when the evoked muscle force exceeds 65% of the maximum muscle power, the number of activated voxels remains flat in the contralateral SMC [42]. Our patients were asked to imagine carrying out the motor task to their greatest ability and reconfirm that they had done so after the MRI examination. Therefore, we assumed that each motor task was performed to the level of maximum muscle force of each subject, thus the number of activated voxels in the motor cortex should theoretically be the same in each subject. In this respect, we can say that the degree of motor tasks was equal among all of the subjects and all of the examinations.

 In this study, slight associated movements were observed in some subjects during the examination. Associated movements are not desired for fMRI examination; however, we could not completely avoid such movements since we examined patients with disturbance of motor function. In patients with cerebral infarction, it is natural that associated movements are observed during the recovery period. Mild associated movements and mirror movements were reported in a previous study on fMRI in patients with cerebral infarction [19]. Associated movements may not be seen in patients with mild symptoms, complete recovery, or complete paralysis; however, we cannot observe process of recovery in patients with severe symptoms. Anyway, brain activation corresponding to slight associated movements should be small. In our study, when associated movements were observed, the corresponding contralateral SMC should have more activated. Therefore, if we could eliminate influence from associated movements, the activation of the SMC of the patients would be reduced compared with that of healthy subjects.

 fMRI is a very useful method of investigating brain function. Various clinical analysis software, such as SPM, is available to cope with analysis of multiple examinations and multiple patients. fMRI provides means for further elucidation of the mechanisms of functional recovery, in addition to its usefulness for study of cognitive function and preoperative brain mapping.

Among fMRI studies, BPI is considered a desired model to investigate plasticity of the brain involving a condition changes only by neural input/output signals. Brain reorganization is believed to occur in two major stages, namely, the inhibition of neural input/output signals due to injury and the recovery of neural input/output signals after operation. Since the brain itself undergoes no organic change, the process of brain reorganization associated with peripheral nerve pathway changes can be clearly studied in patients with BPI. In contrast, in studies of other pathological conditions of the central nervous system, such as cerebral infarction, involve not only the lesion itself but also other chronic biological problems, changes occurring during monitoring prognosis, and pathological changes related to the cause of infarction. As the number of BPI patients is few, investigation based on sufficiently large number of patient is difficult. However, further information regarding the mechanisms of brain reorganization is hoped with more effort through research of fMRI on BPI patients.

## 5. Conclusions

 We studied brain reorganization in BPI patients sequentially using fMRI. In BPI patients with impaired flexion/extension movement of the affected elbow, activation of the SMC contralateral to the affected side decreased at approximately 3 months after injury. And it was even more minimized at one year after injury (approximately 9 months after surgery). Eventually, in accordance with the recovery of elbow function, activation of the SMC contralateral to the affected side is considered to recover. These findings reflect brain reorganization associated with peripheral nervous changes after injury and after operation for functional reconstruction.

## Figures and Tables

**Table 1:** Clinical findings and treatment of 20 patients with brachial plexus injury.

Patient ID	Age	Sex	Injured side	Location of paralysis	MMT score of Bi	Term from injury to surgery (months)	Type of operation	1st fMRI (months after injury)	2nd fMRI (months after injury/after surgery)	3rd fMRI (months after injury/after surgery)	4th fMRI (months after injury/after surgery)
1	27	M	R	C5-7	0	2	ICN3,4 → MC	2	11/8	23/20	35/32
2	18	M	R	Subclavian	0	2	LC-MC, LC-Rad (nerve grafting)	2	11/8	20/17	33/31
3	41	M	R	C5,6 + Subclavian	0	2	C6-MC (nerve grafting)	2	11/8	20/17	34/31
4	34	M	L	Complete	0	5	ICN3,4 → MC	2	14/9	23/18	34/31
5	22	F	R	Complete	1	3	ICN3,4 → MC, ICN5,6 → TD	3	12/8	22/18	
6	22	M	L	C5,6	2	3	Ac → SS, TD → Ax	3	13/10		
7	19	M	R	SS + Ax	5	5	ST-SS, PC-Ax (nerve grafting)	5	14/9	23/17	
8	34	M	R	C5-8	0	2	ICN3,4 → MC ICN5,6 → TD	0	11/9	22/20	
9	20	M	R	Subclavian	0	4	LC-MC, PC-Ax (nerve grafting)	4			
10	21	M	R	Subclavian	3	3	LC-Rad (nerve grafting)	3	13/10		
11	20	M	L	C5-7	0	4	ICN3,4 → MC Ac → SS TD → Ax	3	13/9	24/20	
12	20	M	R	Complete	0	3	ICN3,4 → MC ICN5,6 → TD	3	13/9	24/20	
13	26	M	R	Complete	0	1	ICN3,4 → MC ICN5,6 → TD	0	9/7		
14	21	M	R	C5-7	1	2	LPN → Ax	1	14/11		
15	30	M	L	C5-7	0	2	ICN3,4,5 → MC Ac → SS	1	14/12		
16	18	M	L	Complete	0	3	ICN3,4 → MC ICN5,6 → TD	1			
17	20	M	R	Complete	0	3	ICN3,4 → MC ICN5,6 → TD	2	14/10		
18	22	M	R	Complete	0	2	ICN3,4 → MC ICN5,6 → TD	1	11/9		
19	32	M	L	Complete	0	1	ICN3,4,5 → MC ICN6,7 → TD	1			
20	35	M	R	Complete	0	1	ICN3,4 → MC ICN5,6 → TD	1			

Abbreviations: Ac: accessory nerve, Ax: axillary nerve, Bi: biceps brachii muscle, ICN: intercostal nerves, LC: lateral cord, LPN: lateral pectoral nerve, LPN: lateral pectoral nerve, MC: musculocutaneous nerve, MMT: manual muscle test, PC: posterior cord, Rad: radial nerve, SS: suprascapular nerve, ST: superior trunk, TD: thoracodorsal nerve, and →: nerve transfer.
